# Supplementary material for: Genetic and epigenetic features direct differential efficiency of Xist-mediated silencing at X-chromosomal and autosomal locations
Source: Nat Commun. 2017 Sep 25;8:690. doi: 10.1038/s41467-017-00528-1 (PMC5612955; doi:10.1038/s41467-017-00528-1)
Supplement: Supplementary file 1 — Supplementary Information [file 41467_2017_528_MOESM1_ESM.pdf]

Description of Supplementary Files

File Name: Supplementary Information
Description: Supplementary Figures and Supplementary Tables

File Name: Peer Review File

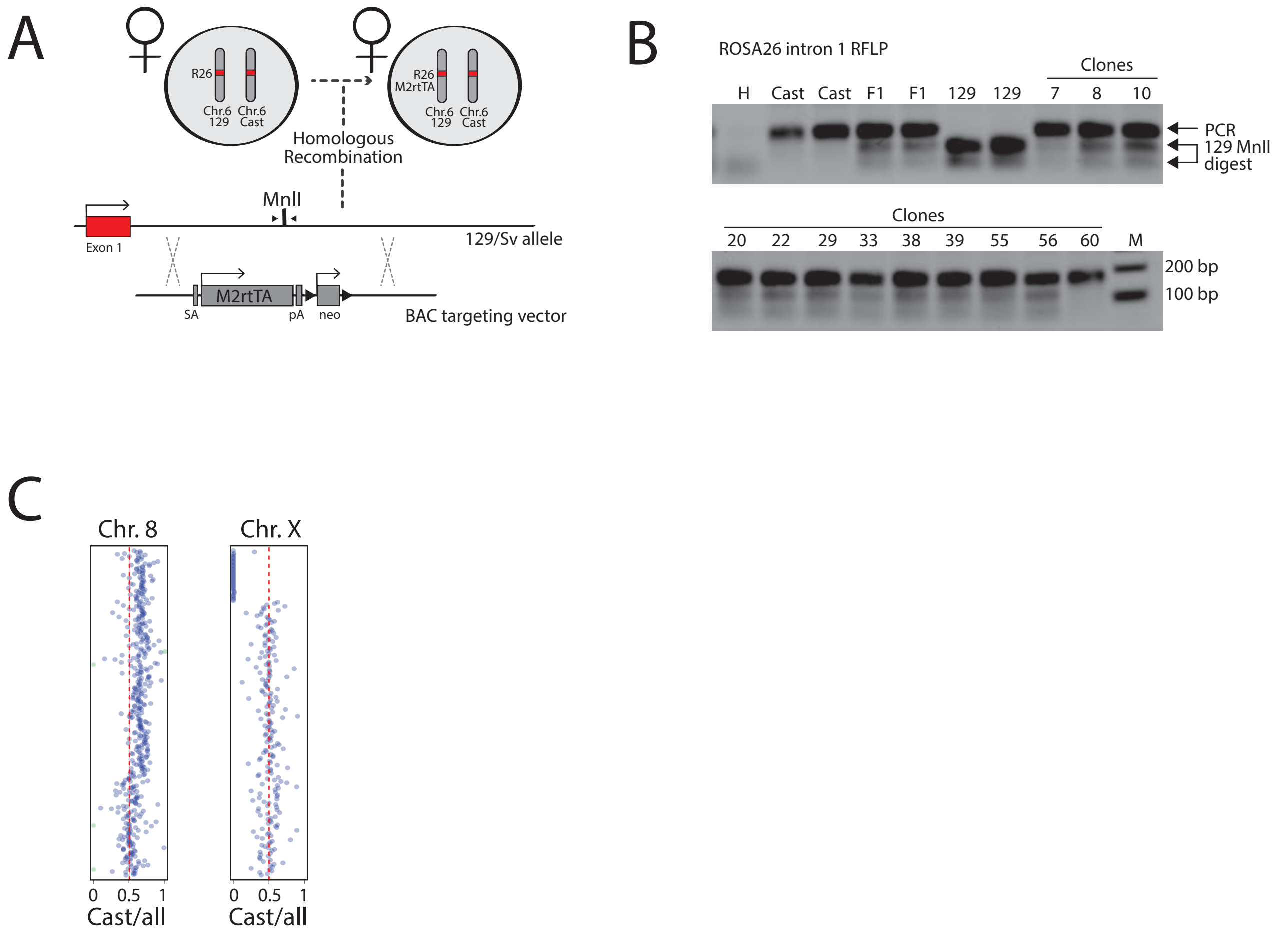

Supplementary Figure 1, Related to Figure 1.

(A)Targeting strategy to generate hybrid F1 2-1 ESC lines constitutively expressing the reverse tetracycline transactivator M2rtTA. M2rtTA has been targeted at the ROSA26 locus together with a neomycin/kanamycin resistance cassette flanked by lox sites. SA, slice acceptor; pA, polyadenylation sequence. (B) PCR-RFLP analysis with primers indicated in Figure S1A spanning a MnlI RFLP discriminating between the Cast/Ei (no MnlI site) and the 129/Sv (MnlI site) alleles, which was used to target the M2rtTA cassette. Correct targeting of the M2rtTA cassette results in loss of 129/Sv specific restriction products, as shown for clone 60. Arrows on right indicate size of PCR product and size of MnlI restriction fragments. F1, F1 2-1 polymorphic 129/Sv-Cast/Ei mother cell line; M, marker; H, water. (C) Mapping of X;8 translocation breakpoint by allele-specific RNA-seq analysis. The distal two-thirds of chromosome 8 are duplicated in the X;8 translocation product whereas 15 Mb of the telomeric end of the 129/Sv X chromosome is lost.

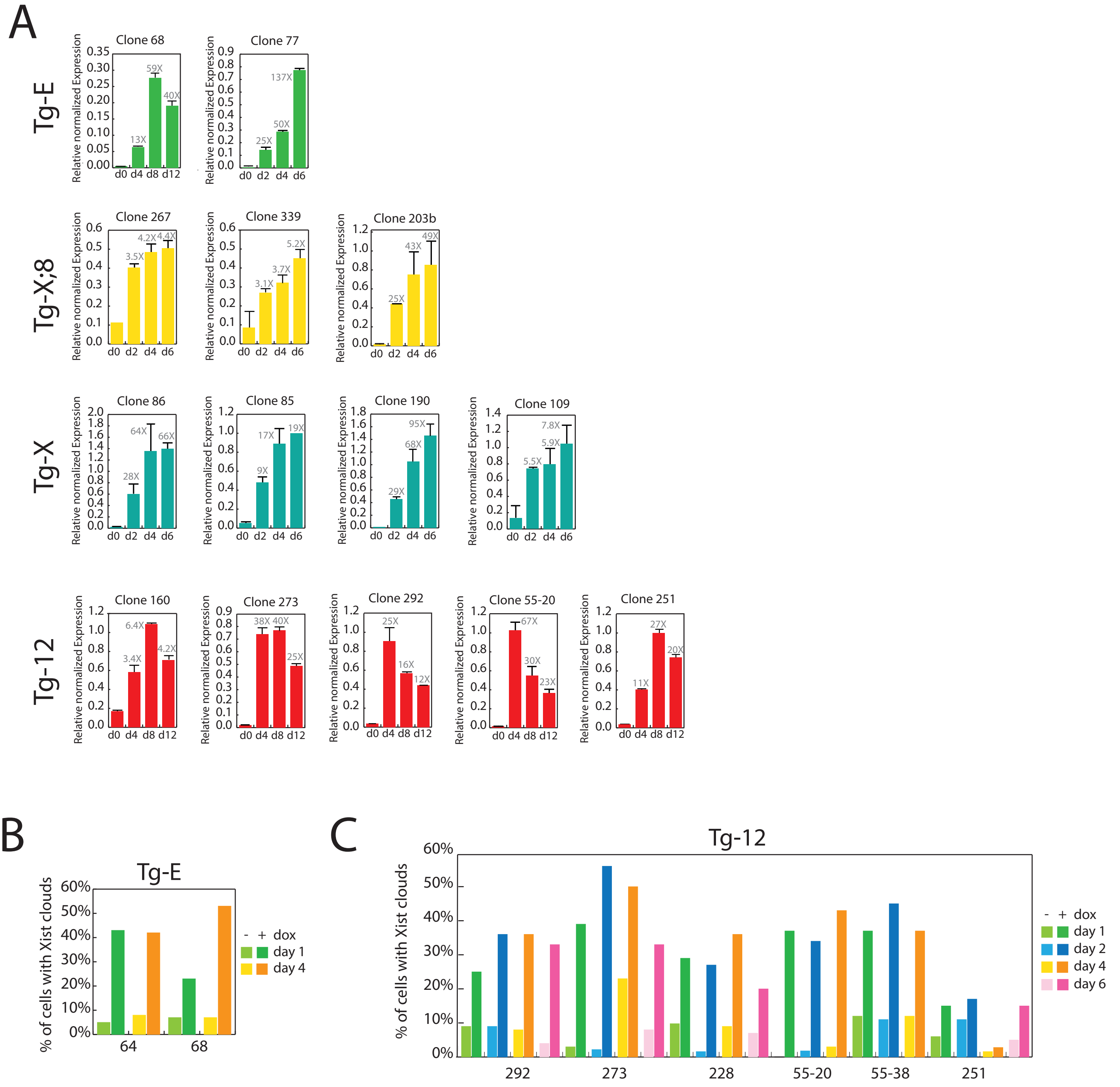

Supplementary Figure 2, Related to Figure 2.
(A) qPCR quantification of Xist RNA expression level in doxycycline untreated cells at different time points upon neuronal differentiation. Mean and SD of two independent differentiation experiments are shown for clones Tg-E (68, 77), Tg-X (86, 85, 190, 109), Tg-X;8 (203b, 267, 339) and Tg-12 (160, 273, 292, 55-20, 251). Fold enrichment relative to day 0 is shown per every tested time point of neuronal differentiation. (B and C) Quantification of cells showing an Xist-coated chromosome at different time points after doxycycline induction. Data related to Tg-E (64, 87) and Tg-12 (292, 273, 228, 55-20, 55-38, 251) clones is shown. n>150 nuclei counted per time point. Higher levels of Xist-coated chromosomes in doxycycline untreated clones compared to Figure 2F is due to serum+Lif culturing conditions used in this experiment versus 2i conditions (Ying et al., 2008) used in Figure 2F.

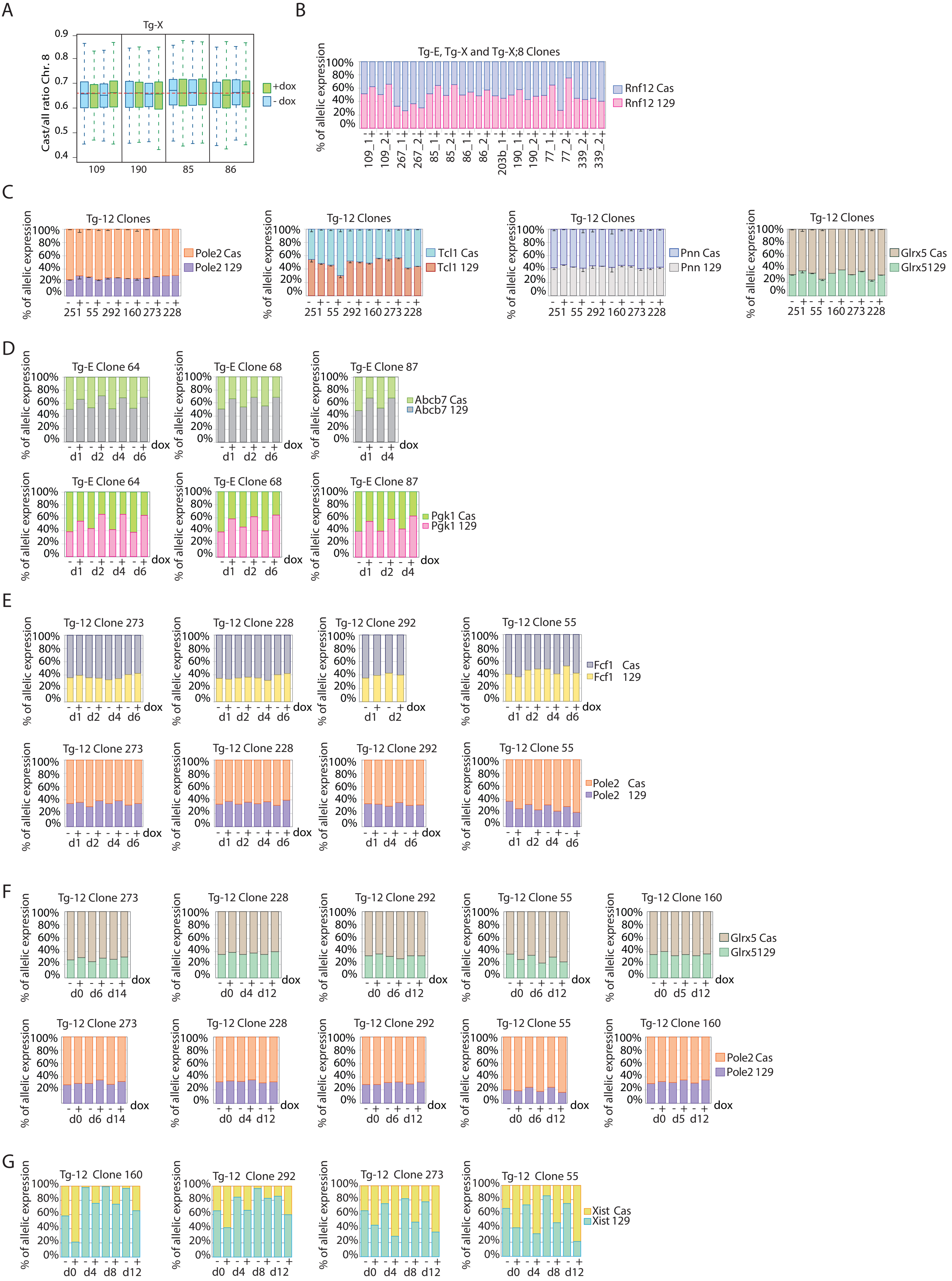

A
Tg-X
Cast/all ratio Chr. 8
+dox
- dox
109
190
85
86
B
Tg-E, Tg-X and Tg-X;8 Clones
% of allelic expression
Rnf12 Cas
Rnf12 129
109_1
109_2
267_1
267_2
85_1
85_2
86_1
86_2
203b_1
190_1
190_2
77_1
77_2
339_2
339_2
C
Tg-12 Clones
Pole2 Cas
Pole2 129
Tcl1 Cas
Tcl1 129
Pnn Cas
Pnn 129
Glrx5 Cas
Glrx5129
251 55 292 160 273 228
251 55 160 273 228
D
Tg-E Clone 64
Tg-E Clone 68
Tg-E Clone 87
Abcb7 Cas
Abcb7 129
Pgk1 Cas
Pgk1 129
dox
d1 d2 d4 d6
E
Tg-12 Clone 273
Tg-12 Clone 228
Tg-12 Clone 292
Tg-12 Clone 55
Fcf1 Cas
Fcf1 129
Pole2 Cas
Pole2 129
d1 d2 d4 d6
d1 d4 d6
F
Tg-12 Clone 273
Tg-12 Clone 228
Tg-12 Clone 292
Tg-12 Clone 55
Tg-12 Clone 160
Glrx5 Cas
Glrx5129
Pole2 Cas
Pole2 129
d0 d6 d14
d0 d4 d12
d0 d6 d12
d0 d5 d12
G
Tg-12 Clone 160
Tg-12 Clone 292
Tg-12 Clone 273
Tg-12 Clone 55
Xist Cas
Xist 129
d0 d4 d8 d12

Supplementary Figure 3, Related to Figure 3.

(A) Box plot showing the Cast- specific gene expression ratio of chromosome 8 genes in Tg-X clones (109, 190, 86, 85). (B and C) Validation of RNA-seq analysis by RT-PCR followed by pyrosequencing of Tg-X (86, 85, 109, 190), Tg-X;8 (203b, 267, 339), Tg-E (68, 87) and Tg-12 (160, 292, 55, 228, 273) clones maintained in 2i conditions. Expression data for *Tcl1*, *Pnn*, *Pole2*, *Glrx5* (Chr.12) and *Rnf12* (Chr.X) are shown. (D and E) RT-PCR analysis followed by pyrosequencing for Tg-E (64, 87, 68) and Tg-12 (273, 228, 292, 55) clones at different time points after Xist induction in ESCs maintained in conventional serum+Lif conditions. Data for *Abcb7*, *Pgk1* (Chr. X), *Fcf1* and *Pole2* (Chr.12) are shown. (F) RT-PCR analysis followed by pyrosequencing for Tg-12 clones (273, 228, 292, 55, 160) at different time points upon neuronal differentiation. Expression data for *Glrx5* and *Pole2* are shown. (G) Xist allele-specific qPCR of Tg-12 clones 160, 292, 273 and 55 at different time points upon neuronal differentiation.

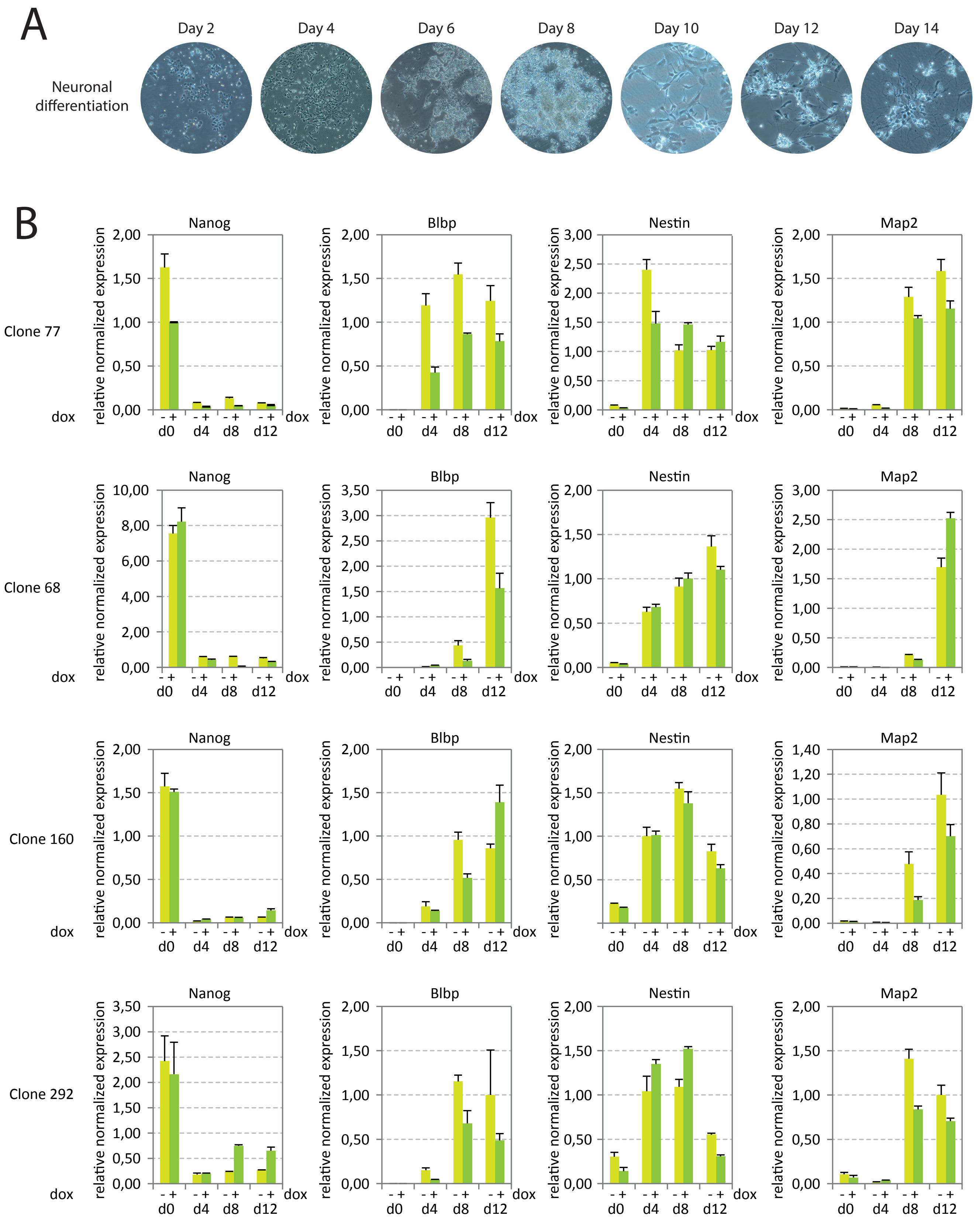

Supplementary Figure 4, Related to Figure 3.
(A) Representative images of *in vitro* neuronal differentiation of F1 2-1 ESC lines. (B) qPCR analysis of Tg-E (77, 68) and Tg-12 (160, 292) clones at different time points upon neuronal differentiation. Data for *Nanog*, *Blbp*, *Nestin* and *Map2* are shown.

A

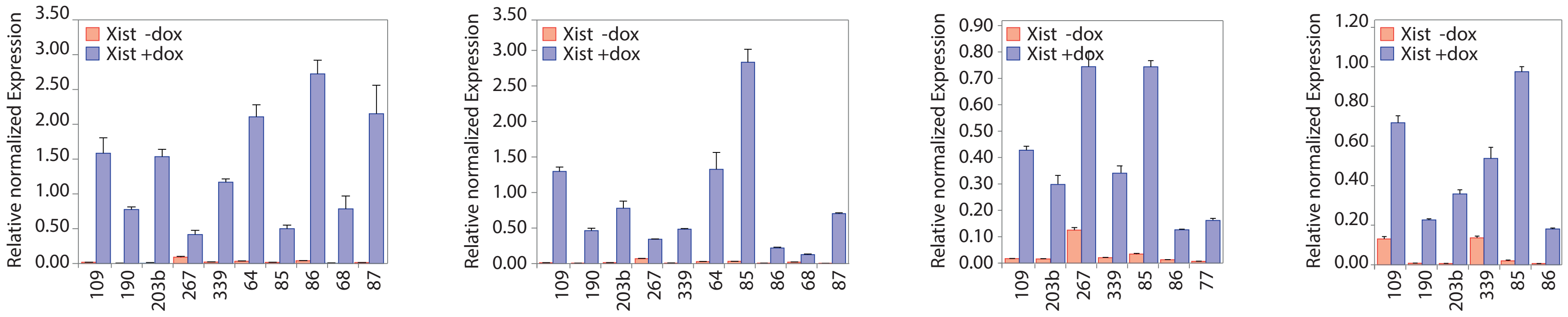

Supplementary Figure 5

(A) Xist qPCR analysis of Tg-E (87,68,77), Tg-X (109, 190, 86, 85) and Tg-X;8 (203B, 339, 267) clones after five days of doxycycline treatment prior to neuronal differentiation. Data of three to four independent experiments per clone are shown

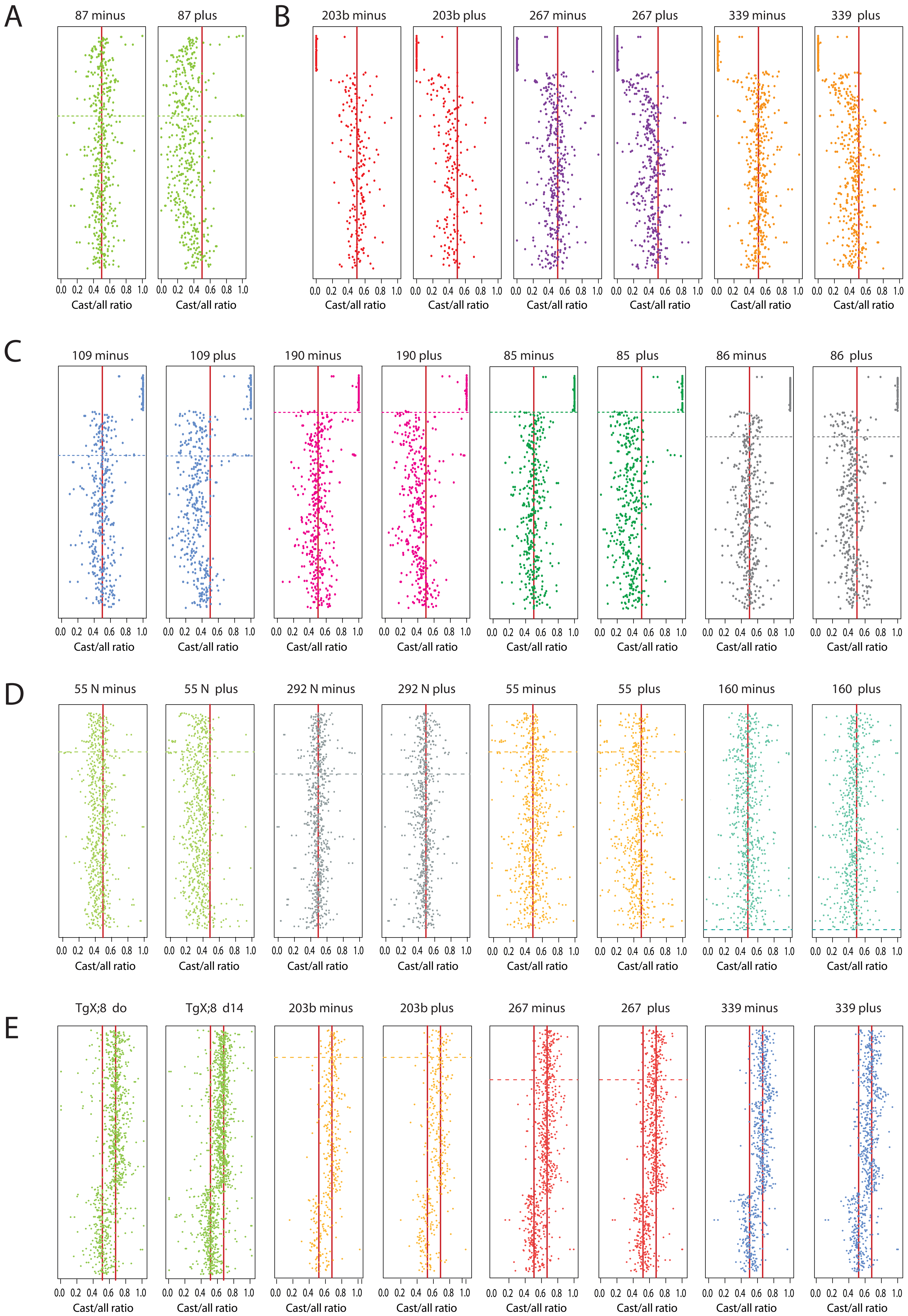

Supplementary Figure 6, Related to Figure 5
(A, B and C) Plots showing changes in Cast/all ratio of X-linked genes upon Xist induction in Tg-E, Tg-X;8 and Tg-X clones. (D and E) Plots showing changes in Cast/all ratio of chromosome 12 and 8 genes in Tg-12 and Tg-X;8 clones. Every dot represents a gene. Genes are ordered by genomic position on chromosomes X from telomeric (top) to centromeric (bottom) ends.

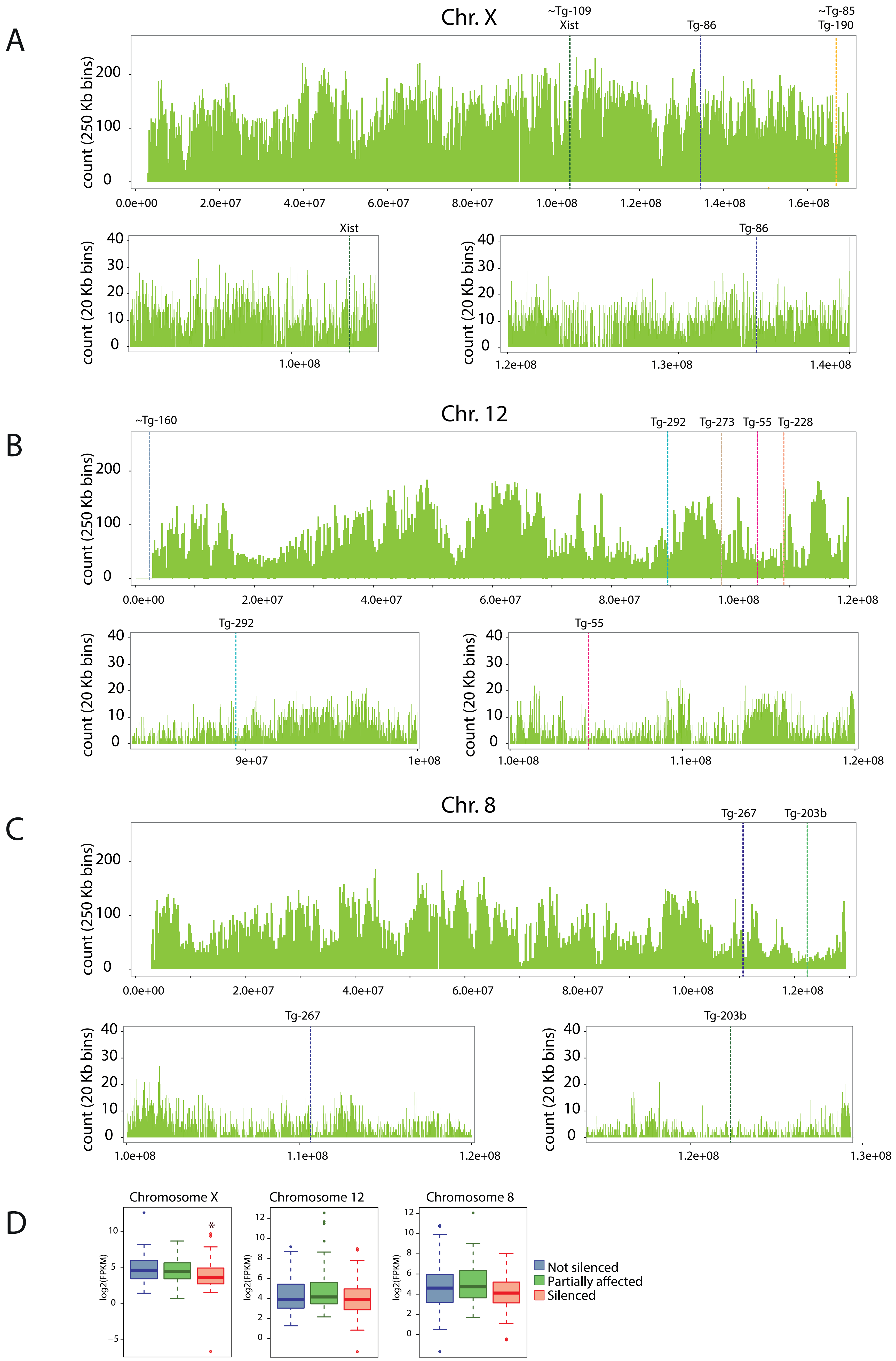

Supplementary Figure 7, Related to Figure 6

LINE density along the entire length of chromosomes X (A), 12 (B), and 8 (C) is shown. Green histogram bars represent 250 kb bins. Zoom in around the integration sites of Xist transgene are shown, histogram bars correspond to 20 kb bins. (D) Box plots showing gene expression of not silenced, partially affected and efficiently silenced X-linked and autosomal genes before ectopic Xist induction. *p<0.05 Wicoxon rank-sum test relative to not silenced genes.

Supplementary Table 1

OLIGO	SEQUENCE	DESCRIPTION
94	ggtaccCAGCCATGTTTGCTCGTTT	PTET XIST_3`ARM_KpnI F
95	ggatccTAAACGCAGGTATCCGAGGT	PTET XIST_3`ARM_BamHI R
96	cactgtcggtcactgttcaga	PTET XIST_5`ARM_F
97	CACACCAAAAGCATCACCAG	PTET XIST_5`ARM_R
98	ctgcagCCCCCTGAACCTGAAACATA	Floxed NEO F
99	ctgcagAAGtctgggtcaagccattg	Floxed NEO R
119	TGGGTCCTTGTTTCTTGACC	PTET XIST 5`BB F k53_BAC_REC
120	TCCCTTTAGGGTTCCGATTT	PTET XIST 5`BB R k53__BAC_REC
121	GCCATCACGAGATTTCGATT	PTET XIST 5`BB R k35__BAC_REC
127	ATCGCCTGGAGAATTCGAG	PTET XIST 3`BB F__BAC_REC
128	CGATGGGCAAAAGAAAAAGA	PTET XIST 3`BB F__BAC_REC
90	CGCGTCATGTCACTGAGCTT	Xist targeting TspeI F__ES
91	CGTTGCACGCCTTTAACTGA	Xist targeting TspeI R__ES
114	ATATAgcggccgcGGGCCTATTCTCAGTCCAG	3`ARM ROSA26 F NotI
115	CGATAGtctagaAGAATGCCATGAGTCAAGCC	3`ARM ROSA26 R XbaI
116	tagactGACGTCtctgggggagtcgttttacc	5`ARM ROSA26 AatII F
117	atctaaGAGCTCtttcgaggtcgatcgaggtc	5`ARM ROSA26 SacI R
136	TGAAAACACAAATGGCGTGT	R26 BB 5`F__BAC_REC
137	gcgaagagtttgtcctcaacc	R26 BB 5`R__BAC_REC
138	caatggcttgacccagaCTT	R26 BB 3`F__BAC_REC
139	ACACACCAGAAGAGGGCATC	R26 BB 3`R__BAC_REC
156	ggagagaggcattcatgggag	R26_targeting_22__ES_REC
157	ctttttgttgatcctttgccttgatcc	R26_targeting_23__ES_REC

Primers used in this study to generate transgenic ES clones as described in the methods section

Supplementary Table 2

OLIGO	SEQUENCE	DESCRIPTION
160	GGATCCTGCTTGAACTACTGC	Forward primer *Xist* expression
161	CAGGCAATCCTTCTTCTTGAG	Reverse primer *Xist* expression
162	AACCCTAAGGCCAACCGTGAAAAG	Forward primer *Actin* expression
163	CATGGCTGGGGTGTTGAAGGTCTC	Reverse primer *Actin* expression
327	AACCAAAGGATGAAGTGCAAGCGG	Forward primer *Nanog* expression
328	TCCAAGTTGGGTTGGTCCAAGTCT	Reverse primer *Nanog* expression
323	AACCTGGAAGCTGACAGACAGT	Forward primer *Blbp* expression
324	TCACAGTTGGTTTGGTCACG	Reverse primer *Blbp* expression
333	AGGCGCTGGAACAGAGATT	Forward primer *Nestin* expression
334	TTCCAGGATCTGAGCGATCT	Reverse primer *Nestin* expression
345	CCTTTTAAAACCGGGAGAGG	Forward primer *Map2* expression
346	AAGGAGAGTGGGCCTGAACT	Reverse primer *Map2* expression
255	GGACCAAAATACACAGATGGCCTA	*Xist*_CAS_Forward_allele_specific
257	CATCATTCCGTCCGGTCAAG	*Xist*_129_Forward_allele_specific
256	CTTGGAAGTCACAGGTGTCCTGTA	*Xist*_Reverse_allele_specific

Primers used in this study for gene expression analysis as described in the methods section.

Supplementary Table 3

OLIGO	SEQUENCE	DESCRIPTION
1	AAAGCCTGTTCATCTTCCAT	Set1 chrx:103468694
2	GTCGCTTATAGTGTTTGCTG	Set1 chrx:103468399
3	AAGACCCTTTAAGACGGTTC	Set2 chrx:103580781
4	GGCTGACTCAAACTAAAGGA	Set2 chrx:103580325
5	TGTATGCACAGTTATAGCCA	Set3 chrx:103683355
6	AGTTTATGTCCATCTCCAGTG	Set3 chrx:103683088
7	AAAGGACCCTAAGAAACCATT	Set4 (pTARBAC2.1:4845)
8	TTCAAATATGTATCCGCTCATG	Set4 (pTARBAC2.1:4566)
9	CTTCAGCTTCAAATATCACCC	Set5 (pTARBAC2.1:7667)
10	CTGAATATTCTCTCTGGGCC	Set5 (pTARBAC2.1:7385)
11	GTTCACGGTGCCCTCCAT	Set6-dsRed
12	CGTGATGCAGAAGAAGACCA	Set6-dsRed

Primers used in this study to map the Xist transgenes integration sites by TLA

Supplementary Table 4

oligos	sequence	description
1_Nampt_F1	CACTTCGGTGGCAGAAATAGAGTA	Chr.12
2_Nampt_R1_bio	TAGCGAGCTCTCTGTGATTGTG	Chr.12
3_Nampt_S1	AGTAGTTACAATAGAGACCA	Chr.12
4_Pole2_F1_bio	GAAACTTCTCCAACACTTGCACTT	Chr.12
5_Pole2_R1	AAGGACGCCATGTTTGTGATT	Chr.12
6_Pole2_S1	CATGTTTGTGATTGTGTCT	Chr.12
7_Fcf1_F1	TTATCATGAGTCCTGCAGAATCG	Chr.12
8_Fcf1_R1_bio	AGTCGTCAGCGTAGGTTCCTT	Chr.12
9_Fcf1_S1	GTCCTGCAGAATCGC	Chr.12
10_Pnn_F1_bio	AGAAGAGAGAAAGCAGGTTGAAAA	Chr.12
11_Pnn_R1	TAGCACGTCTCTCCTCAAACAGTT	Chr.12
12_Pnn_S1	GTCTCTCCTCAAACAGTTC	Chr.12
13_Tcl1_1F	GAGTTTTCTTCTTGGAGCCCAGTG	Chr.12
14_Tcl1_1R_bio	CCACAGGGGAGATGTTGGTACTTA	Chr.12
15_Tcl1_1S	GACATGACTAGCGAACAG	Chr.12
16_Glrx5_F1	TCGGGGCCAGAAAAGCCT	Chr.12
17_Glrx5_R1_bio	CCCGCTTGCTGCCAGGAG	Chr.12
18_Glrx5_s1	GTTGCTGTGATGCTTG	Chr.12
19_Abcb7_R1_bio	AGCCACATCAGGAAATTGAAGTC	Chr.X
20_Abcb7__F1	AATGATTATGTGGCAAGTCACG	Chr.X
21_Abcb7_S1	GGTCTTCCTATATTGTCCA	Chr.X
22_Rnf12_R1_bio	GGAGAATACCGGCAGAGAGATA	Chr.X
23_Rnf12_F1	TGTTGTTCGGAGCCTGAGAT	Chr.X
24_Rnf12_S1	CCTGAGATCTTGATCGAGT	Chr.X

Primers used in this study for RT-PCR amplification followed by pyrosequencing.
